# Colorectal Cancer Arising in a Diverticulum: Report of an Unusual Presentation

**DOI:** 10.1155/crgm/4631169

**Published:** 2026-07-05

**Authors:** Paulo de Carvalho Contu, Daniel de Carvalho Damin, Camila Leal Guimarães, Beatriz Padoin Camilo, Jordy Guimarães Costa, Rafaella Aléssio Naibo, Beatriz Lima Silveira, Marina Ferreira da Luz

**Affiliations:** ^1^ Division of Coloproctology, Hospital de Clínicas de Porto Alegre, Porto Alegre, Rio Grande do Sul, Brazil, hcpa.edu.br; ^2^ Department of Surgery, Medical School, Federal University of Rio Grande do Sul, Porto Alegre, Rio Grande do Sul, Brazil, ufrgs.br

## Abstract

The coexistence of diverticulitis and colorectal cancer is rare but clinically significant. We present an unusual case of an elderly patient with recurrent acute diverticulitis, colonic obstruction due to a sigmoid stenosis, in which an adenocarcinoma originated within a diverticulum. This presentation is particularly challenging because the extramural growth pattern within the wall of the pseudodiverticulum, which lacks the muscularis propria layer, facilitates early transmural spread without a significant intraluminal mucosal mass, leading to an advanced diagnosis more frequently. This case underscores the need for high clinical vigilance and specialized radiologic review in patients with complex diverticular disease and unexplained stenosis.

## 1. Introduction

Colonic diverticulosis is a common condition influenced by modifiable factors, such as diet and lifestyle. Acute diverticulitis occurs in up to 5% of these patients and can lead to complications, including colonic stenosis [1, 2]. Although diverticulitis and colorectal cancer (CRC) share some risk factors, such as chronic inflammation and gut microbiome alterations [1, 3], their coexistence is infrequent but clinically relevant. While the association between diverticulitis and CRC has been explored, the pathophysiological mechanisms underlying this association remain unclear.

In this article, we describe a rare case of CRC arising in the mucosa of a colonic diverticulum in a patient with a history of recurrent diverticulitis and colonic stenosis, in which the approach adopted was curative, despite the challenging characteristics given the peculiar circumstances of the development of an adenocarcinoma originating at the base of a diverticulum.

In this context, the diagnostic process becomes difficult and complex, considering the histological architecture of the colonic pseudodiverticulum, characterized by herniation of the mucosa and submucosa through points of weakness in the intestinal muscular wall. Unlike the intact colon, the pseudodiverticulum lacks its own muscular layer, eliminating an anatomical barrier against neoplastic invasion. Thus, adenocarcinomas originating in this environment can progress transmurally directly to the extramural compartment, reaching T3 stages or higher. This anatomical peculiarity can mimic benign strictures and warrant a more guarded prognosis, given the early spread of the neoplasm to the pericolic area and regional lymph nodes.

## 2. Case Report

An 85‐year‐old woman with hypertension and a history of multiple episodes of acute diverticulitis, all confirmed by abdominal CT scans, presented with chronic abdominal pain. On May 17, 2022, a flexible sigmoidoscopy revealed normal findings up to the rectosigmoid junction. However, the procedure could not be advanced due to a fixed angulation. No biopsies were obtained.

**FIGURE 1:**
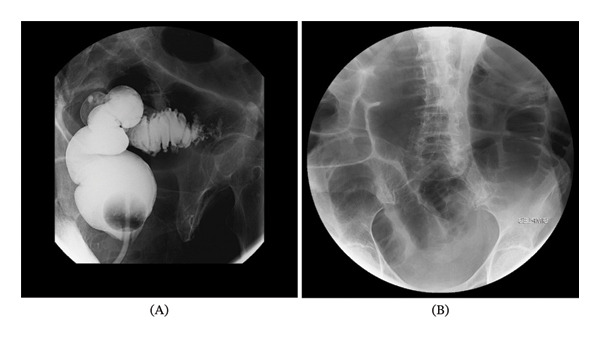
Contrast enema: diverticula and contrast failure at the proximal sigmoid (A) and marked colonic distension (B).

**FIGURE 2:**
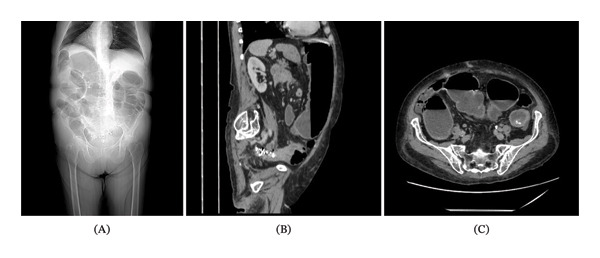
Abdominal CT: intestinal distention (A), obstruction of colonic transit in the sigmoid colon with residual contrast (B), and colonic distension with air–fluid levels (C).

A contrast enema was performed on July 27, 2022, demonstrating diverticula and marked colonic distension, with contrast failure at the proximal sigmoid (Figure 1). On the same day, the patient presented to the Emergency Department with intestinal obstruction. CT imaging revealed concentric thickening of the sigmoid wall, pericolonic fat stranding, residual contrast, obstruction of colonic transit, and intestinal distention (Figure 2).

**FIGURE 3:**
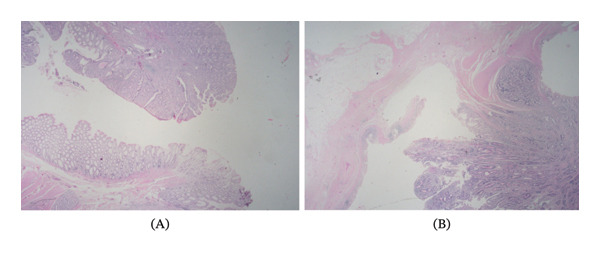
Histopathological images showing the diverticulum and neoplastic transition at its fundus (A and B–40*x*).

The patient underwent an urgent Hartmann’s procedure for colonic obstruction due to stenosis, with intraoperative findings of dense fibrotic infiltration into the mesocolon. There was no macroscopic lymphadenopathy, but malignancy was suspected due to the rigidity of the stenosis and adjacent dense infiltrate. Frozen section analysis of the distal margins was negative for malignancy, but definitive histopathological examination revealed adenocarcinoma arising from a diverticulum (Figure 3), infiltrating the full thickness of the colonic wall and adjacent adipose tissue. Two of 18 resected lymph nodes contained metastases, and immunohistochemical studies confirmed the diagnosis of primary colonic adenocarcinoma.

The postoperative course was uneventful, and, considering the age range, frailty, and the performance status (ECOG 2), it was decided not to perform adjuvant treatment. At the follow‐up, the patient remains asymptomatic with no evidence of recurrence currently.

## 3. Discussion

There is an association between diverticular disease and an increased risk of overall cancer [4]. The simultaneous occurrence of acute diverticulitis and CRC poses a complex diagnostic and therapeutic challenge. Although each condition is common in the elderly population, their coexistence, particularly when the carcinoma arises directly within a diverticulum, is unusual and often under‐recognized in clinical practice.

Both acute diverticulitis and CRC share overlapping risk factors, such as diets low in fiber and high in red or processed meat, physical inactivity, obesity, smoking, and advanced age [2–6]. Chronic inflammation is a central pathogenic mechanism linking these two conditions. Recurrent episodes of diverticulitis trigger a persistent inflammatory milieu characterized by the release of cytokines, the recruitment of immune cells, and the generation of reactive oxygen species (ROS) [4]. ROS induce oxidative stress, causing direct DNA damage and genomic instability, which may facilitate the accumulation of oncogenic mutations. In parallel, repeated mucosal injury can promote abnormal epithelial proliferation and dysplasia, creating a microenvironment conducive to carcinogenesis [7].

Epidemiological data support this association. In a meta‐analysis of 50,445 patients, Meyer et al. [8] found that CRC prevalence was 7.9% among those with complicated diverticulitis, compared with 1.3% among those with uncomplicated diverticulitis, a sixfold increased risk. Mortensen et al. [9] similarly demonstrated a higher long‐term incidence of CRC among patients with a history of diverticulitis compared with matched controls. Furthermore, Sharma et al. [10] concluded that complicated diverticulitis should prompt thorough colonic evaluation, given a reported CRC detection rate exceeding 10% in such cases. Recently published guidelines [11] strongly recommend that colonoscopy or CT colonography should be performed within the first year after an episode of acute diverticulitis to exclude CRC unless colonoscopy was performed recently.

From a diagnostic perspective, cancers arising within a diverticulum pose unique challenges. Since the pseudodiverticulum lacks its own muscularis propria layer, there is no mechanical barrier against transmural invasion, which offers less structural resistance to tumor spread. Unlike intraluminal tumors, these neoplasms may extend extramurally, spreading into pericolic fat or adjacent structures without producing a mucosal mass. This phenomenon can lead to false‐negative colonoscopy results or nondiagnostic imaging findings, as the tumor mass may remain hidden within the diverticular sac or infiltrate the bowel wall from the outside, enabling early transmural invasion and resulting in advanced‐stage disease (T3 or greater) at presentation [12–15]. This explains why our patient already presented with two metastatic lymph nodes.

Several reports highlight the diagnostic pitfalls associated with this presentation. Kayano et al. [12] described a patient initially thought to have a benign colovesical fistula due to diverticulitis. Still, the final histology revealed carcinoma within a diverticulum, necessitating additional surgery for bladder recurrence. Van Beurden et al. [16] reported a patient undergoing resection for presumed inflammatory disease, in whom histopathology unexpectedly revealed adenocarcinoma originating in a diverticulum. Konishi et al. [17] documented a case where colonoscopy failed to detect any mucosal abnormality, yet postoperative examination showed a diverticulum‐based carcinoma in the ascending colon.

Table 1 presents a comparison of cases of adenocarcinoma originating in a diverticulum, demonstrating that endoscopy is often inconclusive [12, 13, 16, 17].

**TABLE 1:** Comparison of cases of adenocarcinoma originating in a diverticulum.

Author (year)	Age, gender	Clinical presentation	Endoscopy	Site	Stage (TNM)
Current case	85, F	Intestinal obstruction	Fixed stenosis	Sigmoid	pT3 N1b M0
Kayano et al. [12] (2019)	78, M	Colovesical fistula	Hidden mass	Sigmoid	pT3 N0 M0
Kikuchi et al. [13] (1999)	65, M	Hidden bleeding	Polyp inside a diverticulum	Sigmoid	pTis
Van Beurden et al. [16] (2008)	67, F	Recurrent diverticulitis	Stenosis	Sigmoid	pT3 N0 M0
Konishi et al. [17] (2019)	71, M	Asymptomatic	Normal	Ascending colon	pT1 N0 M0

Recurrent acute diverticular episodes lead to chronic muscular hypertrophy, wall thickening, and fibrosis of the colon, ultimately resulting in luminal narrowing. This condition may mimic or obscure malignancy [18, 19]. Both conditions, CRC and diverticular disease, may require surgery, but with different surgical approaches. CRC necessitates oncological resection of the affected segment, whereas limited resection of the diseased bowel segment is often sufficient in cases of non‐neoplastic conditions [18].

In this case, the patient’s age, history of multiple episodes of acute diverticulitis, and imaging studies consistent with benign stenosis directed the diagnostic process toward a nonmalignant etiology. Furthermore, the diagnostic challenge was further heightened by the clinical presentation of acute intestinal obstruction. The confounding factor manifested itself in the failure of sigmoidoscopy to identify mucosal lesions since the tumor developed within the diverticulum. Clinical reasoning, from this perspective, must consider that the absence of the muscularis propria layer at the site of tumor origin facilitates direct infiltration of the mesocolon. This phenomenon explains why, despite the radiological appearance of concentric stenosis suggestive of a chronic inflammatory process, histopathological analysis revealed full‐thickness invasion with metastases in two of 18 resected lymph nodes. Nonetheless, surgical decision‐making, combining resection with oncological principles and frozen‐section analysis, proved essential to achieving complete tumor removal. This underscores the importance of maintaining a high index of suspicion for malignancy in elderly patients with recurrent acute diverticulitis and unexplained strictures, even in the absence of clear endoscopic or radiologic evidence.

From a clinical management perspective, these cases underscore the need for individualized strategies. For patients with a history of complicated diverticulitis, especially those with persistent or recurrent symptoms, a comprehensive workup, including colonoscopy, cross‐sectional imaging, and, in select cases, diagnostic laparoscopy, should be considered before definitive surgery. Awareness of this potential association also has implications for follow‐up protocols, as residual diverticula in the remaining colon may theoretically harbor similar malignant potential.

In summary, CRC arising within a diverticulum is a rare but clinically significant entity. Its extraluminal growth pattern, coupled with the anatomical peculiarities of pseudodiverticula, makes diagnosis particularly challenging. Early recognition requires vigilance, careful interpretation of imaging, and sometimes surgical exploration. Our case reinforces the importance of considering CRC in the differential diagnosis of diverticulitis, with a view toward prompt and definitive management to optimize outcomes.

This case highlights an uncommon presentation of CRC arising within a colonic diverticulum, emphasizing the diagnostic difficulties and the need for oncologic vigilance in patients with recurrent diverticulitis and unexplained colonic stenosis. Early recognition may improve outcomes, even in elderly patients.

## Funding

No funding was received for this manuscript.

## Consent

No written consent has been obtained from the patients, as there are no patient‐identifiable data included in this case report.

## Conflicts of Interest

The authors declare no conflicts of interest.

## Data Availability

The data that support the findings of this study are available from the corresponding author upon reasonable request.
